# Bilateral coexistence of keratoconus and macular corneal dystrophy

**DOI:** 10.4103/0974-620X.53037

**Published:** 2009

**Authors:** Ghazi Al-Hamdan, Sultan Al-Mutairi, Eiman Al-Adwani, Abdullah Al-Mujaini

**Affiliations:** 1 Department of Ophthalmology, Al-Bahar Ophthalmology Centre, State of Kuwait; 2 Department of Ophthalmology, Sultan Qaboos University, Muscat, Sultanate of Oman

**Keywords:** Corneal pachymetry, keratoconus, macular dystrophy

## Abstract

Bilateral coexistence of keratoconus and macular corneal dystrophy is a very rare clinical entity. Further elaboration on the possible genetic, histopathologic, pathophysiologic and biochemical correlation is required to study the nature of the condition.

The authors hereby report a 21-year-old female who presented with the typical signs and topographic evidence of keratoconus in association with macular corneal dystrophy. Histopathologic evaluation from the excised corneal button after corneal transplant confirmed the diagnosis.

To our knowledge, there is only one previous report in the literature linking the association of keratoconus and macular corneal dystrophy in the same eye bilaterally.

## Introduction

Worldwide, macular corneal dystrophy is the least common and the most severe among the three classic stromal dystrophies.[1] However, in the Arabian Peninsula, macular stromal dystrophy is seen more often than granular and lattice dystrophies.[2] It is characterized by corneal opacities resulting from intra- and extracellular deposits within the corneal stroma.[3]

Keratoconus, on the other hand, is one of the noninflammatory ectatic diseases in which the cornea assumes a conical shape because of thinning and protrusion that lead to excessive myopia and irregular astigmatism.[4]

To our knowledge, only one case report of simultaneous occurrence of keratoconus and macular corneal dystrophy has been reported in the literature.[5]

## Case Report

A 21-year-old female presented with a history of bilateral gradual reduction of her vision associated with photophobia. Her family history was significant for macular corneal dystrophy; her mother, three sisters, and two brothers, were diagnosed with the same condition.

**Figure 1:**
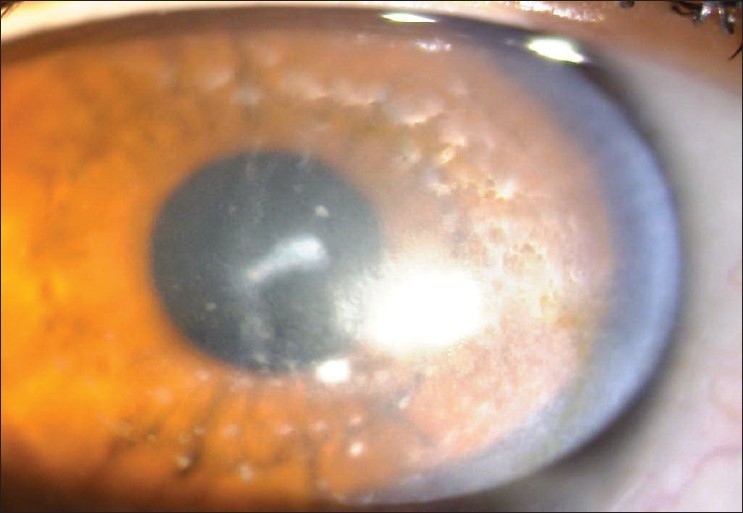
Corneal image of the right eye showing multiple white-grey stromal deposits and Fleisher ring

Ophthalmic examination showed a best-corrected visual acuity (BCVA) of 20/300 and 20/50 in her right and left eye, respectively. External examination, ocular motility, and pupillary reactions were normal. Slit-lamp biomicroscopy revealed focal grayish-white opacities with indistinct borders in both corneas, as well as, presence of Fleisher ring at the base of the cone [Figure 1]. Diffuse haziness extending to the corneal periphery was more severe in the right eye.

**Figure 2:**
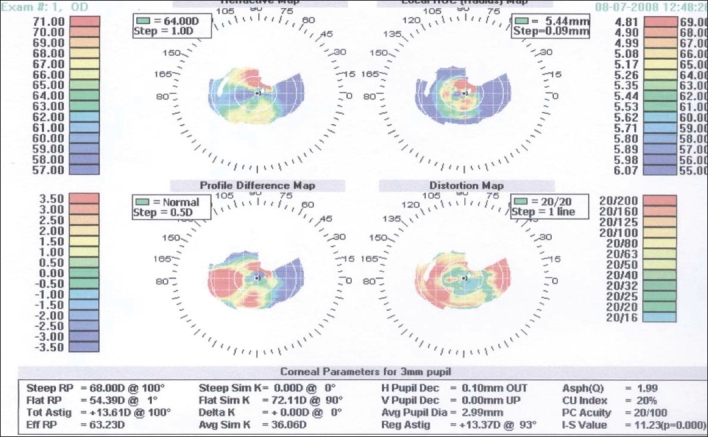
Holladay diagnostic summary of the right eye consistent with advanced keratoconus

**Figure 3:**
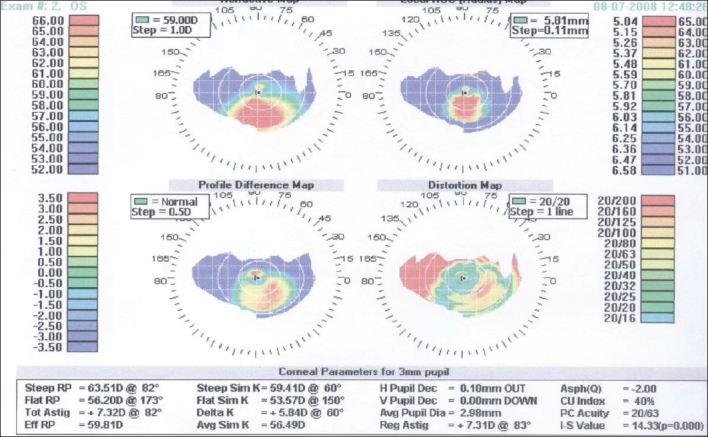
Holladay diagnostic summary of the left eye with moderate to severe keratoconus

Additionally, corneal pachymetry was thin in both eyes (less than 300 µm) with inferotemporal bulge, corneal videokeratography showed irregular mires, and Holladay diagnostic summaries were consistent with keratoconus in both eyes [Figures 2 and 3].

The clinical diagnosis of bilateral concurrent keratoconus with macular corneal dystrophy was done and the patient was advised to have corneal transplant in the right eye.

**Figure 4:**
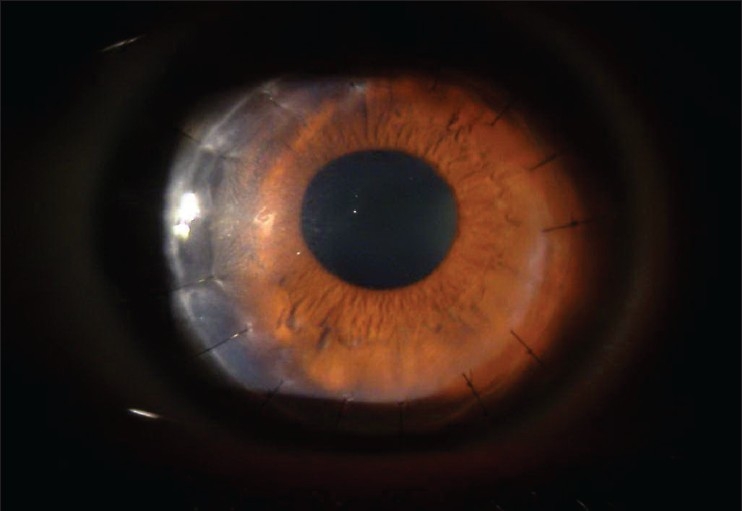
Corneal image of the right eye after deep lamellar keratoplasty

Uneventful deep lamellar keratoplasty was carried out [Figure 4] and the corneal button was sent for histopathology, which was reported as; multiple deposits between collagen fibrils and within keratocytes that stained blue with Alcian blue.

Postoperatively, the patient showed a gradual improvement of the vision.

## Discussion

Usually, the symmetric changes of macular dystrophy are initially noted between 3−9 years of age and are characterized by a diffuse, fine, superficial clouding in the central stroma. By the second decade of life, multiple, irregular, dense, grey-white opacities with indistinct borders extending to the periphery and usually involving the entire thickness of the cornea, are noted.[1] Macular dystrophy, which is usually inherited as an autosomal recessive disease is often associated with reduced corneal thickness and corneal guttata.[6,7]

On the other hand, keratoconus with an estimated prevalence of 50 to 230 cases per 100,000 can occur at any period in life but is more common during adolescence (when stability usually ensues).[1] Although, the hereditary pattern is unpredictable, positive family history can be detected in 6−8% of the patients.[4]

Keratoconus has been reported with numerous corneal dystrophies, but its association with macular corneal dystrophy is extremely rare and has been reported only once.[5] In such a combination (as in our patient), keratoconus could play an additive role for vision reduction. This should be kept in mind in the cases of macular corneal dystrophies with visual deterioration that can not be explained by the amount of corneal stromal deposits and haze.

Although it is documented that central corneal thinning occurs in macular corneal dystrophy,[6] the great majority of patients do not show corneal ectasia. Why ectasia occurs in some thin corneas and does not occur in others is not clear. Both keratoconic corneas and those with macular dystrophy show decreased levels of keratan sulfate and an increased ratio of dermatan to keratan sulfate.[5,8] Based on that, a biochemical alteration in collagen fibril size or packing induced by the abnormal deposits in macular dystrophy may predispose to thinning and ectasia.[5]

In our patient, the typical clinical appearance of the stromal deposits, corneal thinning, and documented histopathology report of the corneal button excludes the possibility of other stromal dystrophies. Additionally, keratoconus was diagnosed clinically based on the typical corneal signs and confirmed by videokeratography.

In conclusion, keratoconus and macular corneal dystrophy can coexist although it is extremely rare. This coexistence needs further studies on the possible genetic, histopathologic, pathophysiologic, and biochemical correlation between the two conditions.
